# ERK-mediated autophagy promotes inactivated Sendai virus (HVJ-E)-induced apoptosis in HeLa cells in an Atg3-dependent manner

**DOI:** 10.1186/s12935-018-0692-y

**Published:** 2018-12-04

**Authors:** Tao Wang, Ning Yu, Miao Qian, Jie Feng, Shuyang Cao, Jun Yin, Quan Zhang

**Affiliations:** 1 Institute of Comparative Medicine, College of Veterinary Medicine, Yangzhou University, 12 Wenhui East Road, Yangzhou, 225009 China; 2 Jiangsu Co-innovation Center for Prevention and Control of Important Animal Infectious Diseases and Zoonoses, Yangzhou University, Yangzhou, 225009 Jiangsu China; 3 Shanghai Laboratory Animal Research Center, Shanghai, 201203 China; 4 College of Medicine, Yangzhou University, Yangzhou, 225009 China

**Keywords:** HVJ-E, Apoptosis, Autophagy, ERK, HeLa cell

## Abstract

**Background:**

Apoptosis and autophagy are known to play important roles in cancer development. It has been reported that HVJ-E induces apoptosis in cancer cells, thereby inhibiting the development of tumors. To define the mechanism by which HVJ-E induces cell death, we examined whether HVJ-E activates autophagic and apoptotic signaling pathways in HeLa cells.

**Methods:**

Cells were treated with chloroquine (CQ) and rapamycin to determine whether autophagy is involved in HVJ-E-induced apoptosis. Treatment with the ERK inhibitor, U0126, was used to determine whether autophagy and apoptosis are mediated by the ERK pathway. Activators of the PI3K/Akt/mTOR/p70S6K pathway, 740 Y-P and SC79, were used to characterize its role in HVJ-E-induced autophagy. siRNA against Atg3 was used to knock down the protein and determine whether it plays a role in HVJ-E-induced apoptosis in HeLa cells.

**Results:**

We found that HVJ-E infection inhibited cell viability and induced apoptosis through the mitochondrial pathway, as evidenced by the expression of caspase proteins. This process was promoted by rapamycin treatment and inhibited by CQ treatment. HVJ-E-induced autophagy was further blocked by 740 Y-P, SC79, and U0126, indicating that both the ERK- and the PI3K/Akt/mTOR/p70S6K-pathways were involved. Finally, autophagy-mediated apoptosis induced by HVJ-E was inhibited by siRNA-mediated Atg3 knockdown.

**Conclusion:**

In HeLa cells, HVJ-E infection triggered autophagy through the PI3K/Akt/mTOR/p70S6K pathway in an ERK1/2-dependent manner, and the induction of autophagy promoted apoptosis in an Atg3-dependent manner.

## Background

Cervical cancer is the third most commonly diagnosed cancer in women globally, and malignant cervical neoplasias are the second most common cause of death among women [1]. Currently, there exist several methods to treat cervical cancer, including surgical therapy [2], gene therapy [3], immunity therapy [4], radiotherapy [5], and chemotherapy [6]. However, tumors can be resistant to certain types of available therapies, including chemotherapy, thereby increasing the difficulty of acquiring sufficient treatment [7]. New therapeutic options are urgently required in order to meet these treatment needs. Oncolytic virus infection has shown great potential as a new cancer treatment method [8], and several oncolytic viruses have been identified and developed as safe and effective therapeutic tools [9]. Presumably, tumors are infected with oncolytic viruses which then lyse and kill the cancerous cell. A previous study has reported that cervical carcinoma cells are sensitive to the vesicular stomatitis virus, and that cells infected with the human papilloma virus are receptive to oncolytic virus therapy [10]. In recent years, inactivated Sendai virus particles (hemagglutinating virus of Japan envelope, HVJ-E) have been shown to contribute to several anti-cancer effects, such as the activation of anti-tumor immunity via anti-tumorigenic neutrophils in the tumor microenvironment [11], the suppression of murine melanoma growth by host immune response, and the down-regulation of beta-catenin expression [12].

Apoptosis is the principal mechanism behind programmed cell death, and apoptosis functions through several complex biochemical and genetic pathways. Apoptosis plays a critical role during the development and aging in normal tissues, which contributes to the healthy balance between cell survival and cell death [13, 14]. Insufficient apoptosis typically results in cancer or autoimmunity, while accelerated cell death is a hallmark of many diseases [15]. Recently, HVJ-E was found to promote apoptosis in various cancer cells, including murine melanoma cells and human prostate cancer PC3 cells [16, 17]. HVJ-E was also found to induce autophagy in human lung cancer cells [18].

Autophagy is reported as a cellular survival strategy that eliminates intracellular proteins and organelles to sustain metabolic balance in cells [19, 20]. However, an increasing pool of evidence indicates that autophagy is a regulated programmed death process, which is closely associated with the development of tumors. It has been demonstrated that autophagy is involved in tumor suppression during the early stages of cancer development [21, 22]. While some models have shown that cancer initiation is suppressed by autophagy, it is also true that autophagy provides nutrients that support the growth of advanced malignant tumors [23, 24]. The exact role of autophagy in tumor cells may be dependent on the type of tumor, the stage of tumorigenesis, or the nature and extent of the insult to the cell [25]. Thus, it is important to clarify the relationship between autophagy and apoptosis as a prelude to tumor suppression.

It has been reported that the PI3K/Akt/mTOR/p70S6K signaling pathway is involved in regulation of the cell cycle, cellular transformation, tumorigenesis, and autophagy during chemotherapy [26, 27]. Moreover, the mitogen-activated protein kinase (MAPK) signaling pathway has been shown to induce autophagy in various cancer cells [28]. The extracellular signal-regulated kinase (ERK) signaling pathway has been identified as a player in the initiation of both autophagy and apoptosis induced by deprivation of amino acids or treatment with aurintricarboxylic acid, β-group soyasaponins, or curcumin [29–31].

Although apoptosis and autophagy can be determined alternatively [26, 27], the question remains as to whether autophagy is induced by a separate death effector mechanism independent of apoptosis, or whether it is a trigger for or dependent upon apoptosis [32, 33]. Autophagy has been shown in some cases to both promote caspase-independent cell death and modulate caspase-mediated apoptosis [34–38]. Moreover, autophagy and apoptosis share common regulators, such as Ca^2+^, reactive oxygen species (ROS), and the presence of endoplasmic reticulum (ER) stress [32, 39]. Studies have shown that ERK activity can promote either intrinsic or extrinsic apoptotic pathways by triggering mitochondrial cytochrome c release or caspase-8 activation [40]. However, it remains unclear whether autophagy is involved in ERK-mediated apoptosis.

Atg12 can modify multiple protein targets in mammalian cells, which has been identified to conjugate to Atg5 in autophagy process. Atg3, an E2-like enzyme that conjugates Atg8 to phosphatidylethanolamine (PE), has been identified as a second target of Atg12 conjugation. The Atg12–Atg3 complex has been found to regulate the early steps of autophagy, and the disruption of the Atg12–Atg3 conjugation has profound effects on mitochondrial function [41]. In this study, we aimed to explore the molecular mechanisms behind autophagy-related apoptosis in cancer cells, which could provide novel insights into developing novel cancer therapies.

## Materials and methods

### Cells, plasmids, and virus

HeLa cells were purchased from the Institute of Biochemistry and Cell Biology in Shanghai, China. GFP-microtubule-associated protein 1 light chain 3 (GFP-LC3) plasmids were kindly provided by Dr. Songshu Meng (Dalian Medical University, Dalian, China). Sendai virus (Z strain) samples were harvested from the chorioallantoic fluid of 10–14 day old chick eggs, purified by centrifugation, and inactivated by UV irradiation (99 mJ/cm^2^), as described previously [42].

### Antibodies and reagents

Antibodies used in this study were purchased from Cell Signaling Technology and included: caspase-3, caspase-9, phospho-mTOR, phospho-Akt, phospho-p70S6K, phospho-JNK, phospho-p38, phospho-ERK1/2, total mTOR, total Akt, total p70S6K, total JNK, total p38, total ERK1/2, Beclin 1, p62, p53, Bcl-2, Bax, Atg3, β-actin, and PARP. The pan-caspase inhibitor Z-VAD-FMK was purchased from Promega and the specific inhibitors of MEK (U0126), The polyclonal rabbit anti-microtubule-associated protein 1A/1B-light chain 3 (LC3) antibody, rapamycin (rap), chloroquine (CQ), and horseradish peroxidase (HRP)-conjugated goat anti-rabbit immunoglobulin were obtained from Sigma-Aldrich. The FITC-Annexin V Apoptosis Detection Kit I used in the study was purchased from BD Bioscience.

### Cell culture and morphological changes

HeLa cells were cultured in Dulbecco’s Modified Eagle Medium (DMEM) supplemented with 10% fetal bovine serum in a humidified cell incubator with an atmosphere of 5% CO_2_ at 37 °C. HeLa cells were treated with the indicated multiplicity of infection (MOI) of HVJ-E for 24 h, and then morphological changes in the cells were photographed using an inverted microscope (DMI 3000B, Leica) at 200× magnification.

### Cell viability assay

Cells were seeded into 96-well plates at a density of 1 × 10^4^ cells per well and incubated for 24 h. C-terminal octapeptide of cholecystokinin (CCK-8) assays (Beyotime, Shanghai, China) were used to assess cell viability after infection with various MOIs. The optical density (OD) at 450 nm was read using a Bio-Tek ELISA microplate reader. The viability rate was calculated as the ratio of the OD in experimental wells to the OD in normal wells.

### Analysis of apoptosis using flow cytometry

Samples containing 5 × 10^5^ cells were treated with HVJ-E for 24 h at the following MOIs: 0, 100, 200, 400, and 800. Cells were harvested and then stained with Annexin V-FITC and PI according to the manufacturer’s instructions. For the apoptosis assays, HeLa cells were incubated with either Z-VAD-FMK inhibitor, rapamycin, or CQ for 40 min, prior to HVJ-E treatment at 800 MOI. The percentage of apoptotic cells was then determined by flow cytometry.

### GFP-LC3 transfection and fluorescence microscopy

The GFP-LC3 plasmids were transfected into HeLa cells using Lipofectamine 2000 (Invitrogen) according to the manufacturer’s guidelines. The formation of GFP-LC3 puncta was observed under a fluorescence microscope (DMI 3000B, Leica) after cells were treated with the rapamycin and HVJ-E as indicated. Cells with five or more puncta were considered to have accumulated autophagosomes, because up to four puncta were observed in a small number of untreated cells. A total of 100 transfected cells were analyzed in each well, and three independent experiments were performed.

### Western blot analysis

HeLa cells were treated with HVJ-E for 24 h. Proteins were extracted from infected and non-infected cells using a cell lysis buffer. Proteins were then separated by 8–15% SDS-PAGE and transferred to a PVDF membrane. The membranes were blocked in 5% non-fat milk for 1 h before incubation with primary antibody (1:1000) overnight at 4 °C. The bound antibody complexes were detected using a chemiluminescence reagent after membranes were incubated with HRP-conjugated IgG secondary antibodies (1:5000).

### Statistical analysis

All studies were performed as three independent experiments. The data are expressed as mean ± SD. Significant variance between groups was determined using one-way ANOVA. Differences of P < 0.05 were considered statistically significant.

## Results

### HVJ-E inhibited cell viability and induced apoptotic cell death in HeLa cells

**Fig. 1:**
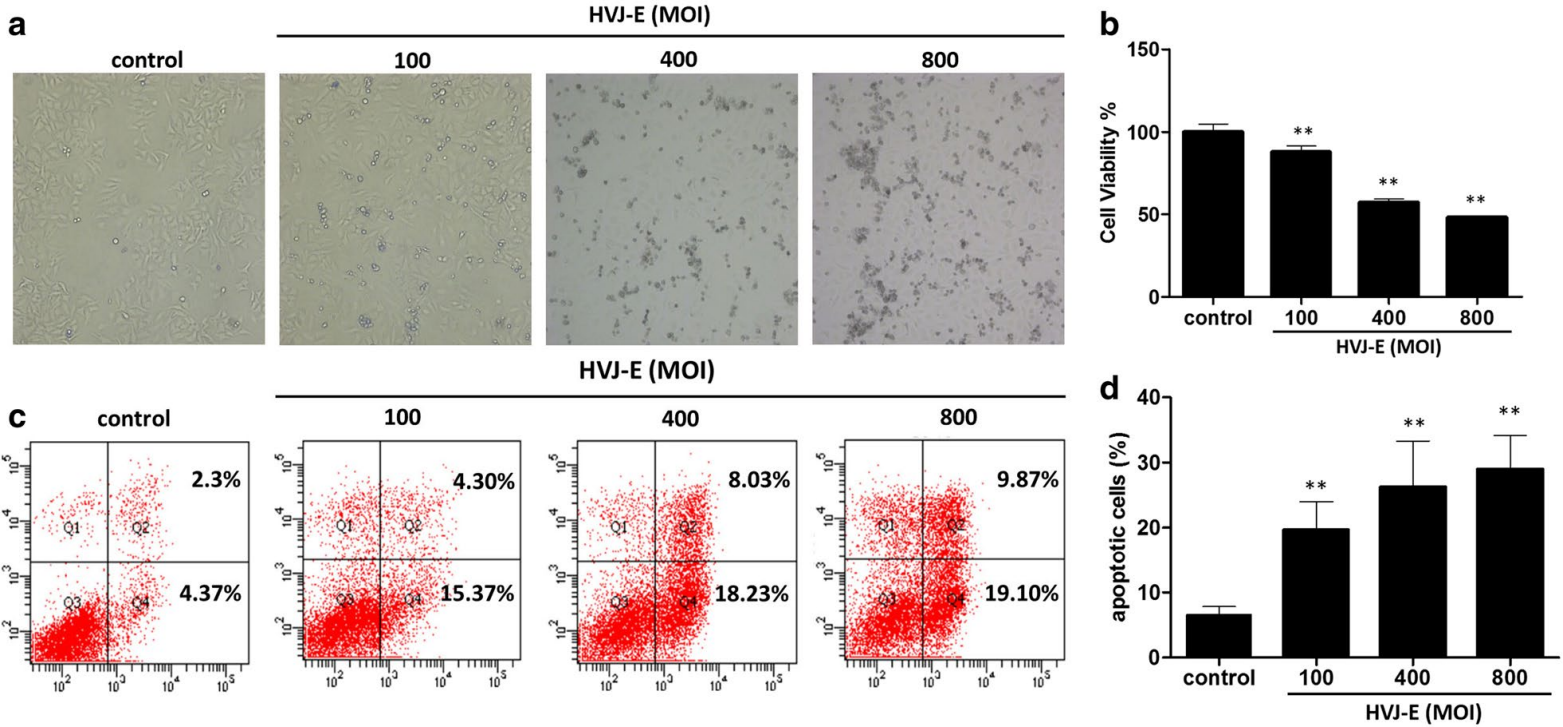
HVJ-E infection inhibited cell viability and induced apoptotic cell death in HeLa cells. HeLa cells were cultured with HVJ-E at the indicated MOI for 24 h.** a** Cell morphology was observed under a microscope.** b** Cell viability was measured by 3-[4,5-dimethylthiazol-2-yl]-2,5-diphenyl tetrazolium bromide (MTT).** c**,** d** Cells were double-stained with Annexin V-FITC (green) and propidium iodide (PI; red) and analyzed by flow cytometry. **P < 0.01

It has been reported that HVJ-E induces apoptosis in some cell types. To determine the effect of HVJ-E on HeLa cells specifically, we first examined cellular viability. As shown in Fig. 1a, infection with either 400 MOI or 800 MOI HVJ-E for 24 h led to obvious changes in cellular morphology and an increase in cell death. Furthermore, HVJ-E significantly decreased the viability of HeLa cells in a dose-dependent manner (Fig. 1b). We hypothesized that this decrease in cellular viability upon HVJ-E treatment was due to the induction of apoptosis. To test this hypothesis, the apoptotic rate was measured by labeling cells with Annexin V-FITC and propidium iodide. As shown in Fig. 1c, d, HVJ-E increased the apoptotic rate in HeLa cells in a dose-dependent manner. The apoptotic rate increased to 19.67% with 100 MOI HVJ-E and to 28.97% with 800 MOI HVJ-E. These results indicate that HVJ-E induces apoptotic cell death in HeLa cells.

### HVJ-E induced mitochondrial apoptosis in HeLa cells in a caspase-dependent manner

**Fig. 2:**
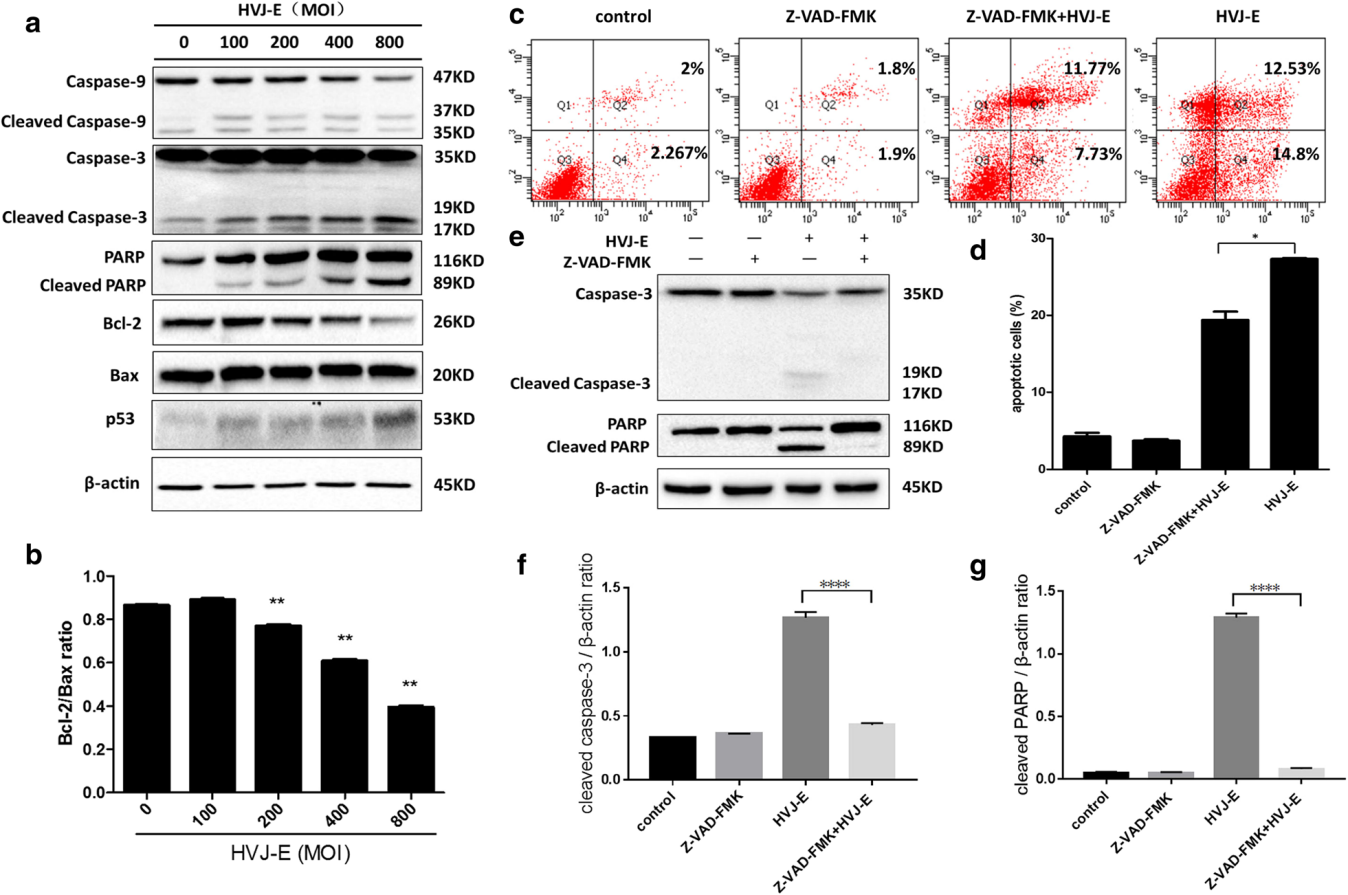
HVJ-E induced mitochondrial apoptosis in HeLa cells in a caspase-dependent manner. HeLa cells were infected with HVJ-E at the indicated MOI for 24 h.** a** Expression levels of cleaved caspase-3, cleaved caspase-9, cleaved PARP, BAX, and Bcl-2 were detected by Western blot. β-Actin was used as a control.** b** Gray value analysis of Bcl-2, Bax, and the ratio of Bcl-2 to Bax.** e**–**g** Cells were treated with the pan-caspase inhibitor, Z-VAD-FMK (50 μM), prior to infection with HVJ-E at 800 MOI. Cleaved caspase-3 and PARP were detected by Western blot. Densities of protein bands were analyzed and normalized to β-actin.** c** Cells were treated with Z-VAD-FMK prior to infection. Cellular apoptosis was assessed and analyzed by flow cytometry. *P < 0.05, **P < 0.01

Mitochondrial dysfunction has been shown to induce apoptotic cell death through the activating caspase-9, caspase-3, and PARP and the increasing the ratio of Bax: Bcl-2, both of which are key steps in apoptosis signaling [23, 43]. To explore the effect of HVJ-E on mitochondrial apoptosis in HeLa cells, we measured the expression levels of the key proteins involved in apoptotic signaling. The expression levels of p53, cleaved caspase-3, cleaved caspase-9, and cleaved PARP were higher, and the ratio of Bax:Bcl-2 was increased in HVJ-E-infected cells compared to controls (Fig. 2a, b). To further confirm that caspases are required for HVJ-E-induced apoptosis in HeLa cells, cells were treated with a pan-caspase inhibitor, Z-VAD-FMK, prior to HVJ-E treatment to inhibit caspase activity. The percentage of apoptotic cells was determined by flow cytometry and protein expression levels were assessed by Western blot. Cells treated with the inhibitor had significantly decreased apoptotic rates compared to controls (Fig. 2c, d), as well as decreased levels of cleaved caspase-3 and cleaved PARP (Fig. 2e–g). These data suggest that HVJ-E-induced apoptosis via the mitochondrial pathway in HeLa cells is caspase-dependent.

### HVJ-E induced autophagic flux in HeLa cells

**Fig. 3:**
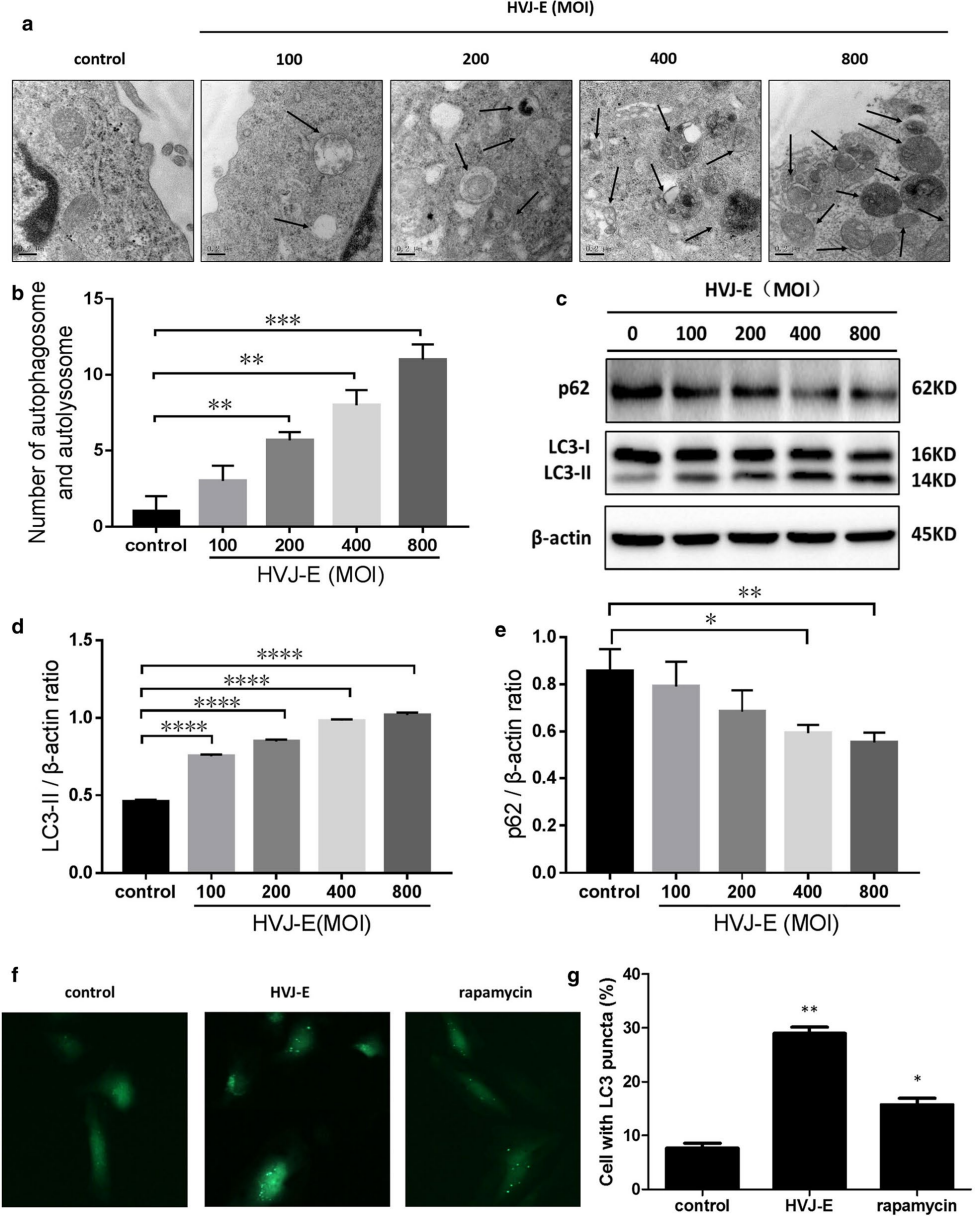
HVJ-E induced autophagic flux in HeLa cells. HeLa cells were cultured with HVJ-E at the indicated MOI.** a** Autophagomes were observed by TEM. β-Actin was used as a control.** b** The statistical number of autophagomes was analyzed.** c**–**e** Expression levels of LC3 and P62 were measured by Western blot was used as a loading control.** f**,** g** HeLa cells were transfected with the GFP-LC3 plasmid and infected with HVJ-E at 800 MOI for 24 h. Rapamycin treatment was used as a positive control. *P < 0.05, **P < 0.01

Autophagy can be observed in cells by visualizing the ultrastructural features of autophagosomal vacuoles by transmission electron microscopy (TEM). As shown in Fig. 3a, b, autophagic vacuoles were rarely observed in electron micrographs from the control group. In contrast, cells from the HVJ-E infection group showed some double-membrane vacuoles with autophagic content. LC3-II and p62—markers of the autophagic process—were also detected in HVJ-E treated cells. As shown in Fig. 3c–e, there was a dose-dependent increase in LC3-II expression and a decrease in p62 expression in HVJ-E-treated cells. Furthermore, there were significantly more GFP-LC3 puncta in HVJ-E-treated cells than in the control cells (Fig. 1f, g). Taken together, these data show that HVJ-E induced autophagic flux in HeLa cells.

### HVJ-E induced autophagy in HeLa cells in a PI3K/Akt/mTOR-dependent manner

**Fig. 4:**
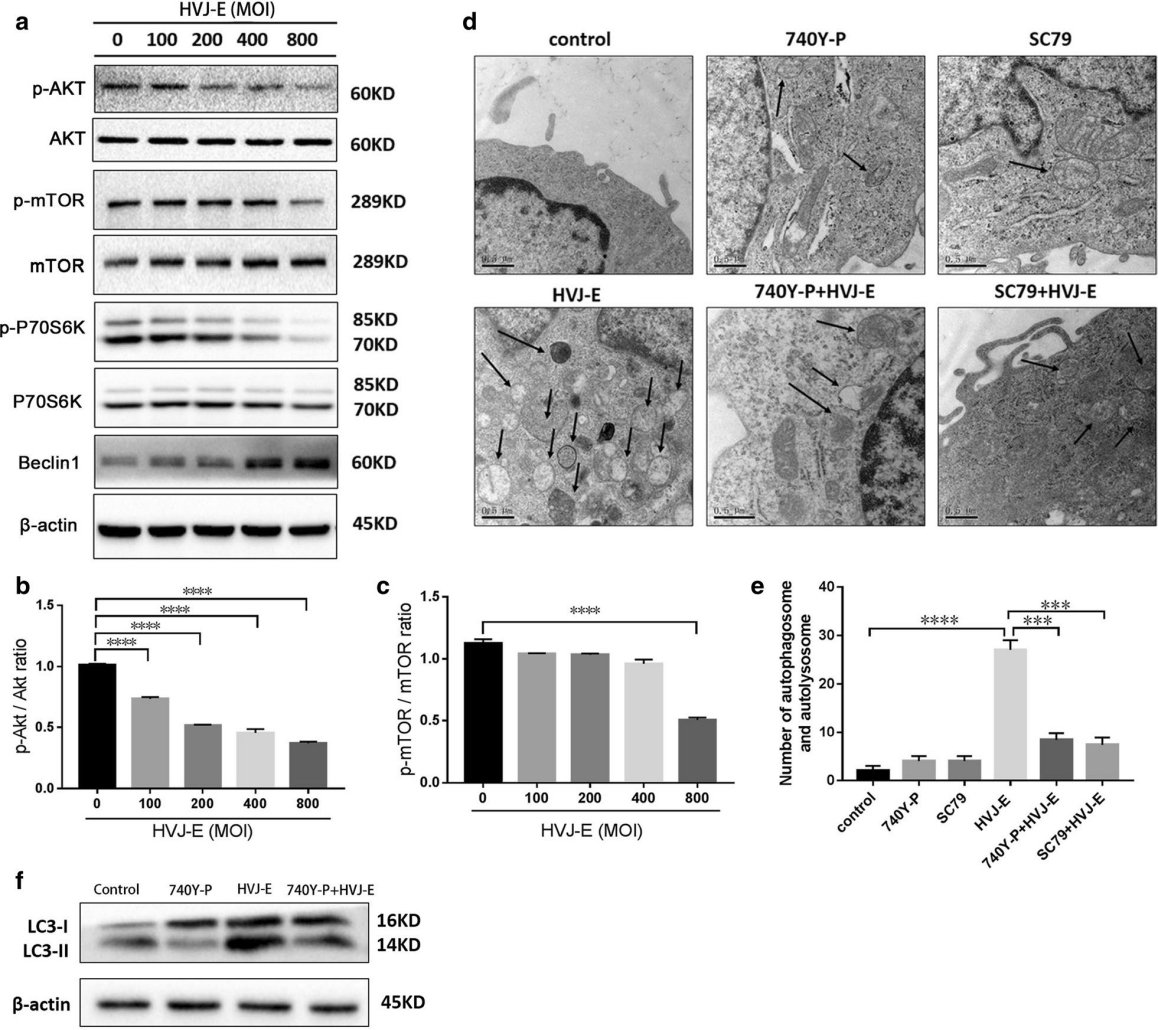
HVJ-E induced autophagy in HeLa cells in a PI3K/AKT/mTOR-dependent manner. HeLa cells were cultured with HVJ-E at the indicated MOI.** a**–**c** Expression levels of p-mTOR, p-70S6K, Akt, and Beclin 1 were detected by Western blot.** d**,** e** Cells were pretreated with 740Y-P or SC79 prior to infection. Autophagosomes in cells were detected by TEM.** f** Cells were pretreated with 740Y-P prior to infection. The expression of LC3 was assessed by Western blot. β-actin was used as a loading control. *P < 0.05, **P < 0.01

The mammalian target of rapamycin (mTOR) is a major negative regulator of autophagy and a downstream target of the phosphatidylinositol 3 kinase (PI3K) and Akt pathways. mTOR is activated by receptors of growth factors, and it stimulates cell growth and differentiation, as well as bolsters cellular resistance to apoptotic signals [44]. To determine whether PI3K/Akt/mTOR signaling is involved in HVJ-induced autophagy, we treated HeLa cells with different MOI HVJ-E and observed the expression levels of key proteins in the pathway. Untreated cells were used as control. As shown in Fig. 4a–c, HVJ-E inhibited the expression of p-AKT, p-mTOR, and p-P70S6K and increased the expression of Beclin 1 in a dose-dependent manner. To directly determine whether these pathways are involved in HVJ-E-mediated autophagy, cells were treated with either 740 Y-P, the pharmacologic activator of mTOR, or SC79, an activator of PI3K and Akt, following HVJ-E treatment. The distribution of autophagosomes was observed by TEM following treatment. As shown in Fig. 4d, e, the number of autophagosomes in cells pretreated with 740 Y-P or SC79 was decreased compared to the control. Furthermore, 740 Y-P treatment inhibited the expression of LC3-II in HVJ-E-treated cells (Fig. 4f). Taken together, these data show that HVJ-E-induced autophagy in HeLa cells is PI3K/AKT/mTOR-dependent.

### ERK1/2 regulates HVJ-E-induced autophagy by inhibiting the PI3K/AKT/mTOR pathway

**Fig. 5:**
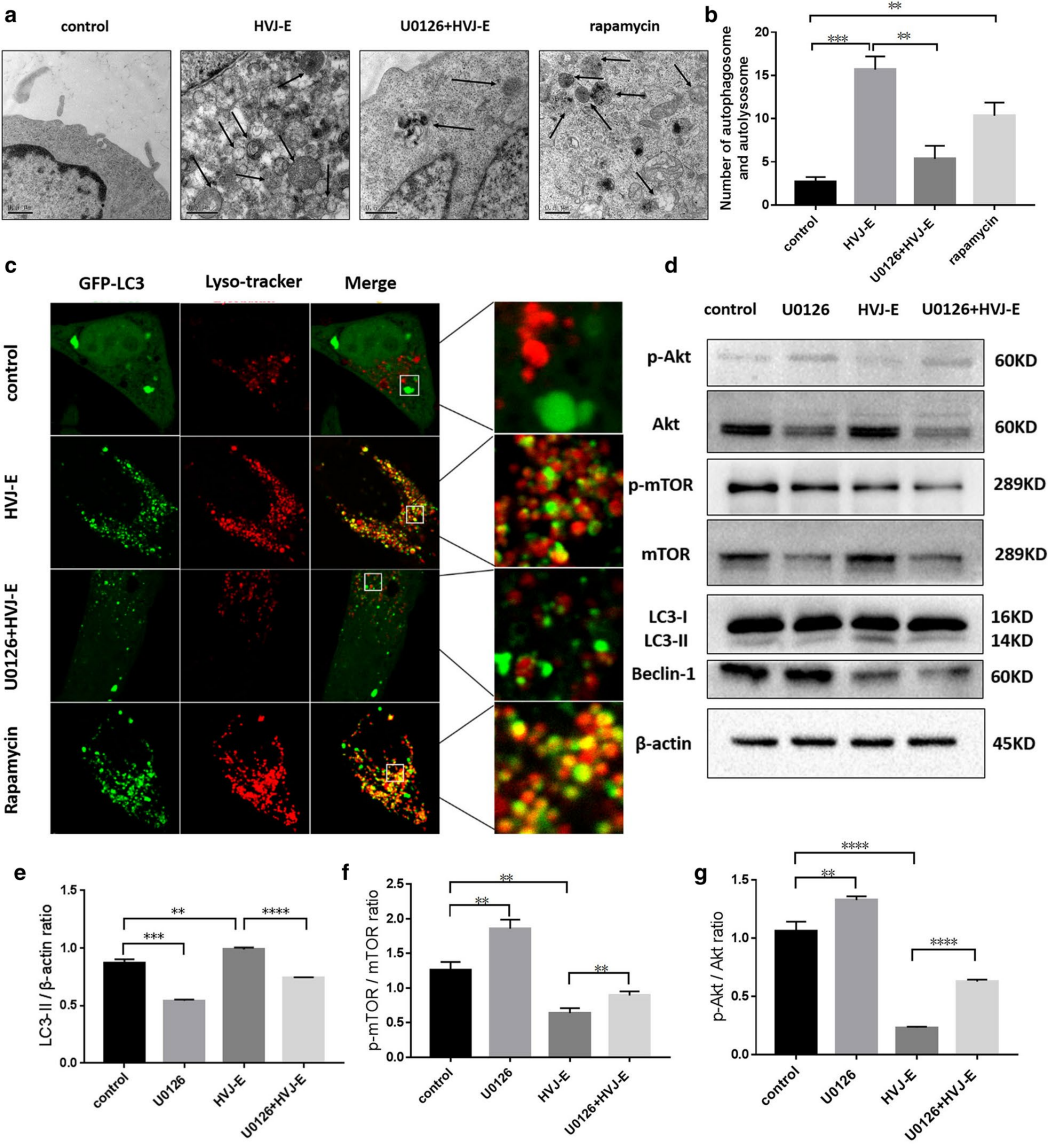
ERK1/2 regulates HVJ-E-induced autophagy by inhibiting the PI3K/AKT/mTOR pathway. HeLa cells were cultured with HVJ-E (800 MOI) in the presence or absence of U0126 (18 μM).** a**,** b** Autophagosomes were detected by TEM. The number of autophagosomes was analyzed according to the distribution in the cells.** c** Cells were stained with lysotracker-red, and the distribution of GFP-LC3 and lysosomes was observed by confocal microscopy.** d**–**g** The phosphorylation of Akt and mTOR, the conversion of LC3, and the expression of Beclin 1 were assessed by Western blot. β-actin was used as a loading control. *P < 0.05, **P < 0.01

Several studies have demonstrated that autophagy is associated with the ERK1/2 signaling pathway [45]. To explore whether ERK1/2 is involved in autophagy induced by HVJ-E infection, cells were treated with U0126, an ERK inhibitor, prior to HVJ-E treatment. Untreated cells were used as controls. TEM revealed that HVJ-E-induced autophagomes were not present in cells pretreated with U0126 (Fig. 5a, b). Furthermore, there was a decrease in co-localization between GFP-LC3 and lysosomes in cells treated with U0126 compared to the control (Fig. 5c). To determine whether ERK1/2 affected autophagy through the regulation of the PI3K/AKT/mTOR pathway, the expression levels of the proteins in U0126-treated and untreated cells were observed. As shown in Fig. 5d–g, U0126 treatment induced the phosphorylation of both AKT and mTOR and decreased the expression of LC3 and Beclin 1. The results indicate that ERK1/2 regulates HVJ-E induced autophagy in HeLa cells by inhibiting the PI3K/AKT/mTOR pathway.

### HVJ-E-induced apoptosis was promoted by the ERK-autophagy pathway in HeLa cells

**Fig. 6:**
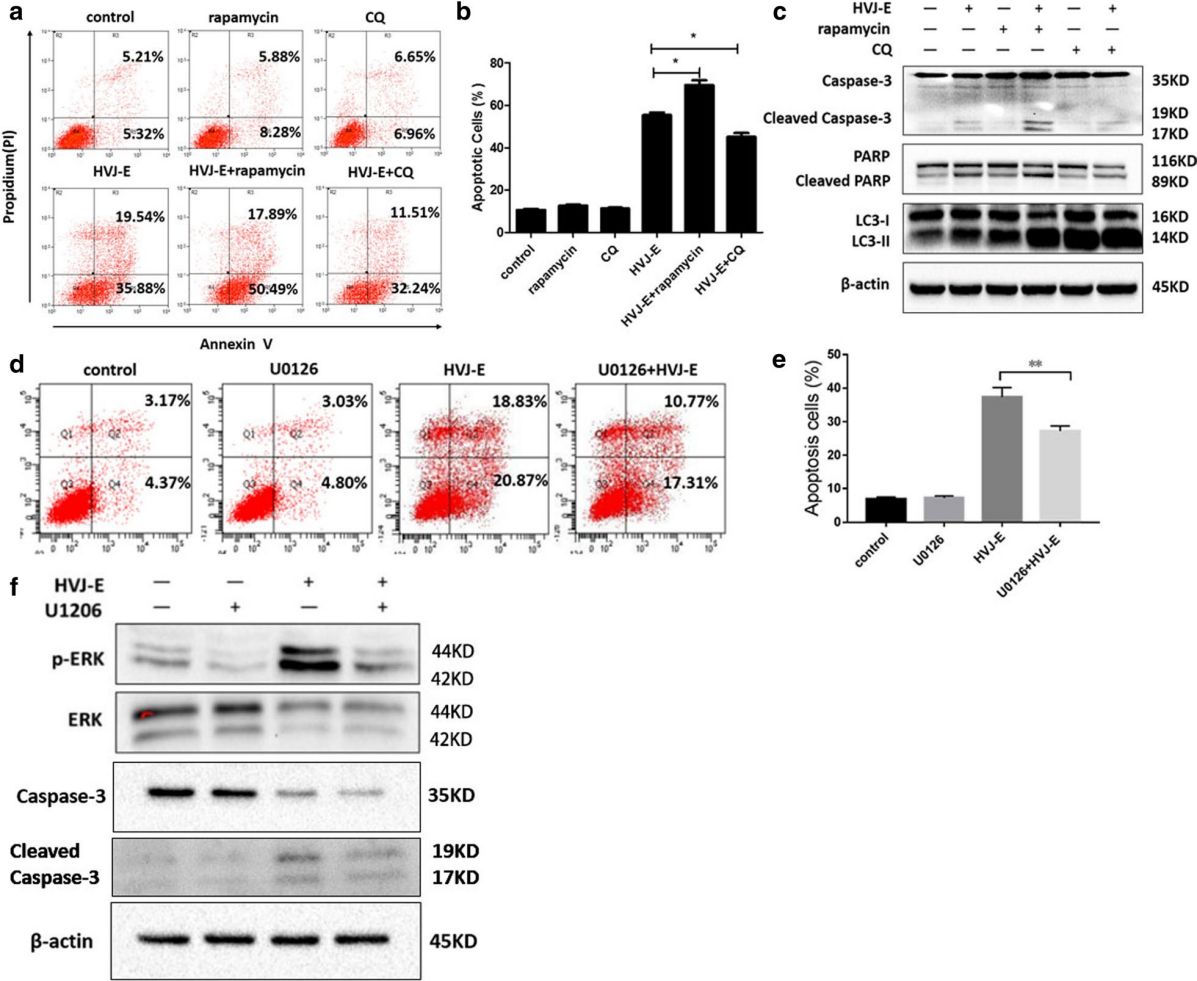
HVJ-E-induced apoptosis was promoted by the ERK-autophagy pathway in HeLa cells. HeLa cells were infected with HVJ-E at the indicated MOI.** a**,** b** Cells were pretreated with rapamycin or chloroquine (CQ) prior to HVJ-E infection. The rate of apoptosis was tested by flow cytometry.** c** Cells were treated as described above. The expression levels of cleaved caspase-3, PARP, and LC3-II were measured by Western blot.** d**,** e** Cells were pretreated with or without U0126 prior to HVJ-E infection. The apoptotic rate was determined by flow cytometry.** f**,** g** Cells were treated as described above. The phosphorylation of ERK and the cleavage of caspase-3 were measured by Western blot. β-Actin was used as a loading control. *P < 0.05, **P < 0.01

To elucidate a biological link between apoptosis and autophagy, HeLa cells were treated with either the autophagy inducer rapamycin (rap) or the autophagy inhibitor chloroquine (CQ) prior to HVJ-E treatment. Untreated cells were used as a control. As shown in Fig. 6a, b, rap pretreatment enhanced apoptosis levels, while CQ decreased apoptosis levels in treated cells compared to the control. Furthermore, LC3 conversion from LC3-I to LC3-II was detected in treated cells. LC3-II levels are higher in infected cells treated with rap than in the control, and the expression of cleaved caspase-3 and cleaved PARP was decreased (Fig. 6c). These data suggest that rapamycin treatment promoted HVJ-E-induced apoptosis via autophagy.

To investigate whether the ERK-autophagy signaling pathway is involved in apoptosis induced by HVJ-E, cells were treated with U0126 to inhibit ERK1/2. As shown in Fig. 6d, e, pre-treatment with U0126 resulted in a reduction of apoptosis induced by HVJ-E and a decrease in cleaved-caspase-3 expression levels (Fig. 6f, g). Taken together, these results show that HVJ-E-induced apoptosis was promoted by the activation of the ERK-autophagy pathway in HeLa cells.

### HVJ-E-induced apoptosis was inhibited by Atg3 knock down in HeLa cells

**Fig. 7:**
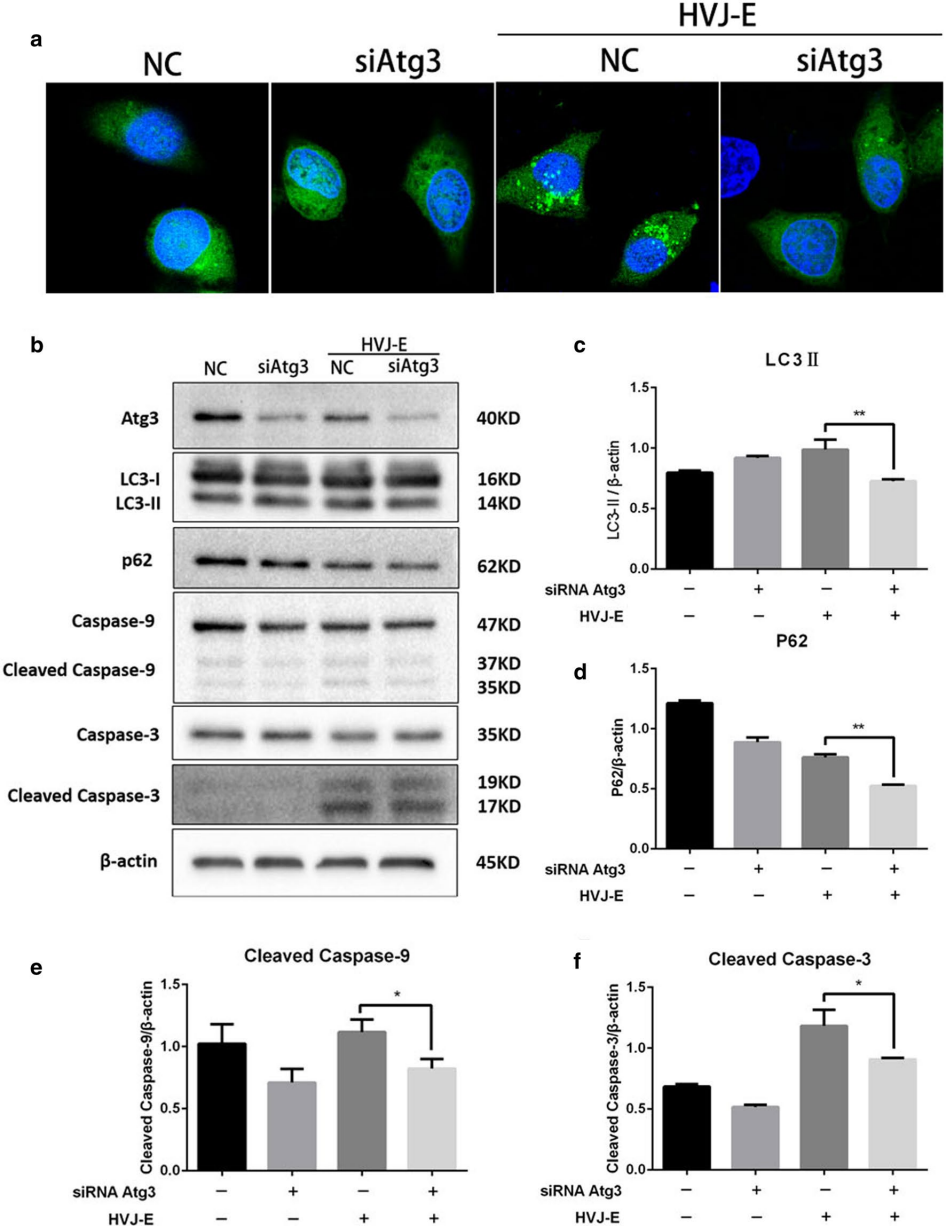
HVJ-E-induced apoptosis was inhibited by Atg3 knock down in HeLa cells. HeLa cells were transfected with Atg3 siRNA prior to infection with HVJ-E(800 MOI).** a** The distribution of GFP-LC3 was detected by confocal microscopy.** b**–**f** The expression levels of Atg3 and p62, the conversion of LC3, the cleavage of Caspase-9, and the cleavage of Caspase-3 were measured by Western blot, β-actin was used as a loading control

Atg3 has been reported to induce mitochondria-mediated apoptosis. To determine whether Atg3 plays a role in autophagy-induced apoptosis in HVJ-E treated cells, siRNA against Atg3 (siAtg3) was used to decrease the expression of the protein in HeLa cells. HVJ-E-infected cells transfected with non-target siRNA were used as controls. As shown in Fig. 7a, the number of LC3 puncta was decreased in cells transfected with siAtg3 compared to control cells. Moreover, the expression of Atg3, the conversion of LC3, and the cleavage of Caspase-9 and Caspase-3 was decreased in cells transfected with siAtg3 (Fig. 7b–e). These data indicate that Atg3 plays a role in HVJ-E-induced apoptosis in HeLa cells.

## Discussion

HVJ-E infection has been shown to activate host immune response [18] and promote apoptosis in cancer cells [46]. It is therefore feasible that HVJ-E could be a medium for cancer treatments. Studies have shown that apoptosis induced by HVJ-E infection is associated with the activation of caspase-8 in PC3 human prostate cancer cells [47] and the activation of caspase-9 in murine B16F10 melanoma cells [12]. However, it was also found that cell death in human neuroblastomas is due to necrosis [48] rather than apoptosis [49]. Based on this evidence, it seemed that whether HVJ-E infection induces apoptosis or necrosis is dependent on the cell type. In this study, we found that HVJ-E induces apoptosis in HeLa cells via the caspase-dependent mitochondrial pathway. We also showed for the first time that autophagic activation promotes apoptosis induced by HVJ-E. Our results demonstrate that, in HeLa cells, HVJ-E induces autophagy through the activation of the ERK pathway, which subsequently leads to the induction of apoptosis via Atg3.

Regulation of autophagy is thought to be a powerful therapeutic strategy in the treatment of various cancers. In fact, many anti-cancer drugs and naturally occurring compounds are reported to have anti-tumor effects brought on by promoting autophagy-dependent apoptotic cell death or senescence in various types of cells. Studies have shown that autophagic flux was blocked in senescent mesenchymal stromal cells (MSCs), indicating that autophagy is closely linked to senescence [50]. Moreover, microRNA (miRNA)-494 induced senescence in human lung cancer cells while suppressing the development of tumor [51]. Which indicates that cell senescence plays an important role in anti-cancer therapy Like cellular senescence, apoptosis is an extreme response to cellular stress, and it represents an important tumor-suppressive mechanism. Our results show that HVJ-E induces apoptosis rather than senescence in HeLa cells, indicating that the fate of cells depends entirely on cell type and their ability to cope with stress. However, further study is needed to identify whether tumor development can be suppressed by HVJ-E infection in vivo.

Despite the existence of of several studies characterizing HVJ-E, there is yet little known about the mechanisms by which HVJ-E induces autophagy in HeLa cells. The current understanding of autophagy is that its induction can result in either cytoprotection or cell death. We have shown that both apoptosis and autophagy are induced by HVJ-E in HeLa cells and that the two are linked. We found that apoptosis was enhanced by rapamycin treatment and inhibited by CQ treatment in HVJ-E-infected HeLa cells. This data indicates that autophagy may act as a death mechanism. This result is consistent with a study performed in A549 cells, wherein it was suggested that inducing autophagy enhances apoptosis, and conversely, inhibition of autophagy suppresses apoptosis triggered by HVJ-E. Interestingly, 3-methyladenine (3-MA), a known inhibitor of autophagy suppressed autophagy and effectively increase the rate of apoptosis in HeLa cells. Therefore, autophagy may play two distinct and opposite roles in apoptosis.

ERK1/2 has been identified as the regulator of apoptosis in many cell types [52, 53]. Cagnol and coworkers found that ERK activity promoted either intrinsic or extrinsic apoptotic pathways and autophagic vacuolization [40]. In our study, we found evidence suggesting that the ERK1/2 signaling pathway induces autophagy in HVJ-E-infected HeLa cells, which subsequently leads to cell death. Many studies have reported that the PI3K/AKT/mTOR pathway mediates the induction of autophagy and apoptosis. Saiki et al. reported that inhibition of the PI3K/AKT/mTOR pathway promotes caffeine-induced apoptosis by enhancing autophagy levels [54]. We have found that HVJ-E-induced autophagy regulated by ERK1/2 is PI3K/AKT/mTOR pathway-dependent. Furthermore, we found the expression of p53 to be activated. Several studies have shown that ERK-mediated p53 expression is required for apoptosis [40].

Recently, JNK activation has been reported to contribute to autophagy and apoptosis via the regulation of Beclin 1/Bcl-2 interaction [55]. Detailed studies of the role this pathway plays in cell death could contribute to its use in novel therapeutic strategies in the future. Studies have also shown that Atg3 activation contributes to apoptosis through regulation of the mitophagy pathway [41, 56]. In this study, we found that both autophagy levels and the rate of mitochondria-mediated apoptosis induced by HVJ-E infection are decreased by knocking down Atg3. This suggests that HVJ-E-induced apoptosis is promoted by autophagy via Atg3. However, we cannot exclude the possible involvement of mitophagy in the protective mechanism. Thus, the exact level of autophagy and whether other apoptotic pathways—including those upstream of apoptosis—are activated require further investigation.

## Conclusion

The results of this study show that HVJ-E infection induces apoptosis in HeLa cells through the mitochondrial pathway, and that this induction is triggered by PI3K/Akt/mTOR/P70SK-mediated autophagy in an ERK-dependent manner. Moreover, the induction of autophagy in HeLa cells promotes HVJ-E-mediated apoptosis, and the inhibition of autophagy protects cells from apoptosis. These findings provide a molecular basis for understanding HVJ-E-mediated cell death and support the notion that combination treatment using an autophagy enhancer is an effective strategy to augment the cytotoxic effects in HeLa cells. These results provide new insight into the mechanisms behind the anti-tumor effects of HVJ-E infection.
